# Editors’ Note

**DOI:** 10.5195/ijt.2024.6683

**Published:** 2025-01-15

**Authors:** Ellen R. Cohn, Jana Cason

**Affiliations:** IJT Co-Editors-in-Chief

## Journal Overview

The International Journal of Telerehabilitation (IJT) is a biannual journal dedicated to advancing telerehabilitation by disseminating peer-reviewed information about current research and practices. IJT is indexed by PubMed and Scopus. IJT is published via the open journal system (OJS) and sponsored by the University of Pittsburgh's Office of Scholarly Communication and Publishing at the University Library System. This institutional support enables IJT to be subscription-free and require no author fees. IJT has recently acquired a second source of institutional sponsorship: Hawai‘i Pacific University, Graduate College of Health Sciences sponsors the effort expended by Dr. Jana Cason as well as this issue's indexing fees.

## International Readership and Authors

IJT enjoys a diverse international audience due to the expanding global relevance of telemedicine, telehealth, and telerehabilitation. Recently, we are pleased to publish work by authors from Australia, Brazil, Canada, Colombia, Egypt, Germany, India, Indonesia, Malaysia, the Netherlands, Poland, Saudi Arabia, Taiwan, Thailand, Ukraine, and the United States. We hope you enjoy reading this new issue of IJT!

## Acknowledgements and New Beginnings

We are grateful to our diligent and generous peer reviewers who donate their time and hail from multiple disciplines. Their suggestions invariably elevate the work of our most experienced authors. Our reviewers are not named herein, to preserve the anonymity of reviews.

Special gratitude is due to recognize both IJT's current support and beginnings. Richard L. Hoover, Interim Director of the Office of Scholarly Communication and Publishing at University of Pittsburgh Library System (ULS), and his staff, have generously and expertly supported this issue and many prior issues. In 2008, Joseph K. Ruffing designed the first IJT journal website and article template. We are grateful to both. Their work will continue to be foundational for IJT.

An exciting new beginning is imminent!. IJT will be “rehomed” to Hawai‘i Pacific University in mid-January 2025. All former issues, and the next issue (slated for April 2025) will be located at: telerehab.hpu.edu The journal will open for new submissions on or about January 20, 2025.

Respectfully,

Jana Cason, DHSc, OTR/L, FAOTA

Ellen R. Cohn, PhD, CCC-SLP, ASHA-F

IJT Co-Editors-in-Chief

